# Corneal Reconstruction with EGFP-Labelled Limbal Mesenchymal Stem Cells in a Rabbit Model of Limbal Stem Cell Deficiency

**DOI:** 10.3390/ijms24065431

**Published:** 2023-03-12

**Authors:** Julia I. Khorolskaya, Daria A. Perepletchikova, Kirill E. Zhurenkov, Daniel V. Kachkin, Aleksandr A. Rubel, Miralda I. Blinova, Natalia A. Mikhailova

**Affiliations:** 1 Institute of Cytology Russian Academy of Science, 194064 St. Petersburg, Russia; 2 Laboratory of Amyloid Biology, St. Petersburg State University, 199034 St. Petersburg, Russia; 3 Department of Genetics and Biotechnology, St. Petersburg State University, 199034 St. Petersburg, Russia

**Keywords:** GFP-labeled cells, ocular cell therapy, cornea regeneration, regenerative ophthalmology, limbal mesenchymal stem cells

## Abstract

Ocular surface reconstruction is essential for treating corneal epithelial defects and vision recovery. Stem cell-based therapy demonstrates promising results but requires further research to elucidate stem cell survival, growth, and differentiation after transplantation in vivo. This study examined the corneal reconstruction promoted by EGFP-labeled limbal mesenchymal stem cells (L-MSCs-EGFP) and their fate after transplantation. EGFP labeling allowed us to evaluate the migration and survival rates of the transferred cells. L-MSCs-EGFP seeded onto decellularized human amniotic membrane (dHAM) were transplanted into rabbits with a modeled limbal stem cell deficiency. The localization and viability of the transplanted cells in animal tissue were analyzed using histology, immunohistochemistry, and confocal microscopy up to 3 months after transplantation. EGFP-labeled cells remained viable for the first 14 days after transplantation. By the 90th day, epithelialization of the rabbit corneas reached 90%, but the presence of viable labeled cells was not observed within the newly formed epithelium. Although labeled cells demonstrated low survivability in host tissue, the squamous corneal-like epithelium was partially restored by the 30th day after transplantation of the tissue-engineered graft. Overall, this study paves the way for further optimization of transplantation conditions and studying the mechanisms of corneal tissue restoration.

## 1. Introduction

The cornea is a transparent outer layer of the eye that performs protective functions and is involved in refracting light. A pool of limbal epithelial stem cells (LESCs) provides corneal regeneration and resides within the limbal region, a narrow ring of tissue between the cornea and conjunctiva [1]. These cells are essential for corneal epithelial tissue repair and physiological regeneration, and prevent the migration of the conjunctival epithelial cells over the corneal surface [2,3]. Limbal mesenchymal stem cells (L-MSCs) are normally adjacent to the basal membrane within the limbal stroma. They are responsible for generating and maintaining the LESC niche [4,5,6].

Severe damage to the limbus and corneal stem cells can lead to limbal stem cell deficiency (LSCD) syndrome. Neovascularization of the ocular surface, corneal opacity, conjunctivalization, chronic inflammation, pain, and visual loss are the most detrimental outcomes of this condition [7,8,9]. It is not always possible to restore corneal functionality through therapeutic and surgical approaches. The major concern in LSCD treatment is the repopulation of the damaged limbal stem cell niche [7]. Nowadays, tissue engineering and stem cell-based therapy may become the most promising treatment for LSCD [10]. Developing limbal and corneal tissue-engineered grafts for restoring the cornea’s functionality and transparency is an actual challenge in regenerative biomedicine. LESCs and L-MSCs are considered the most promising cell types capable of addressing this issue.

Various approaches have been described for isolating and culturing LESCs in vitro and designing various tissue-engineered grafts [11]. However, the shortage of donor materials significantly restricts the broad clinical application of LESCs.

Mesenchymal stem cells (MSCs) have great potential for regenerative medicine due to their high plasticity, self-renewal ability, effective proliferation in vitro, and genetic stability [6,12]. MSCs can be isolated from many tissues, such as bone marrow, adipose tissue, and corneal stroma [13]. Due to the limited number of cells (LESCs) for treating LSCD, MSCs from various sources, including the limbal stroma, have been investigated for corneal epithelial repair [5,11,14,15,16,17]. Holan et al. demonstrated that MSCs had a similar therapeutic outcome to LESCs in a rabbit model of LSCD [18]. Tissue-specific MSCs have a higher regenerative potential than MSCs of other origins. Therefore L-MSCs seem more effective in corneal epithelial repair [6,19]. L-MSCs have been shown to express similar stemness markers to LESCs, such as ABCG2, ABCB5, PAX6, and p63a [4,11,20,21,22]. The plasticity of these cells and the possibility to differentiate into various directions have been previously shown [23]. L-MSCs have a low immunogenic profile and immunosuppressive properties. They can stimulate the proliferation of LESCs in vitro and can be considered a cell component in the development of corneal substitutes [24].

Since there is little information on the participation of transplanted MSCs in the processes of corneal epithelium restoration in vivo, further research is needed to define the mechanisms of their therapeutic properties and to understand their potential side effects.

**Figure 1:**
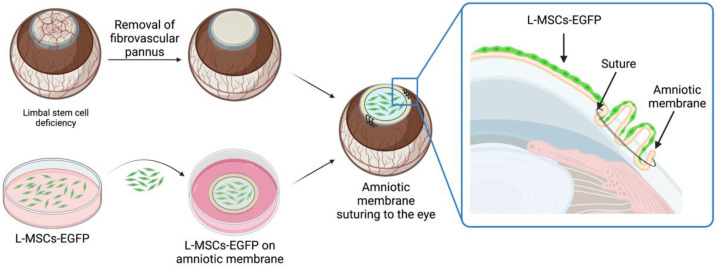
Schematic representation of the tissue-engineered graft preparation and transplantation. Before the transplantation, the fibrovascular pannus was removed from the corneal surface. Decellularized human amniotic membrane (dHAM) with cultured L-MSCs-EGFP was placed on the de-epithelialized corneal stroma with the cells facing up and sutured to the episclera. dHAM was folded across the whole limbus to provide an additional physical barrier and delay conjunctivalization of the cornea after surgery. Created with BioRender.com.

The current work demonstrates the fate of transplanted EGFP-labeled limbal mesenchymal stem cells (L-MSCs-EGFP) in the rabbit model of LSCD. The localization and viability of the transplanted cells in animal tissue were analyzed using histological evaluation up to 3 months after transplantation. Schematic representation of the tissue-engineered graft preparation and transplantation is presented in Figure 1.

## 2. Results

### 2.1. Corneal Reconstruction with L-MSCs-EGFP in the Rabbit Model of Total LSCD

**Figure 2:**
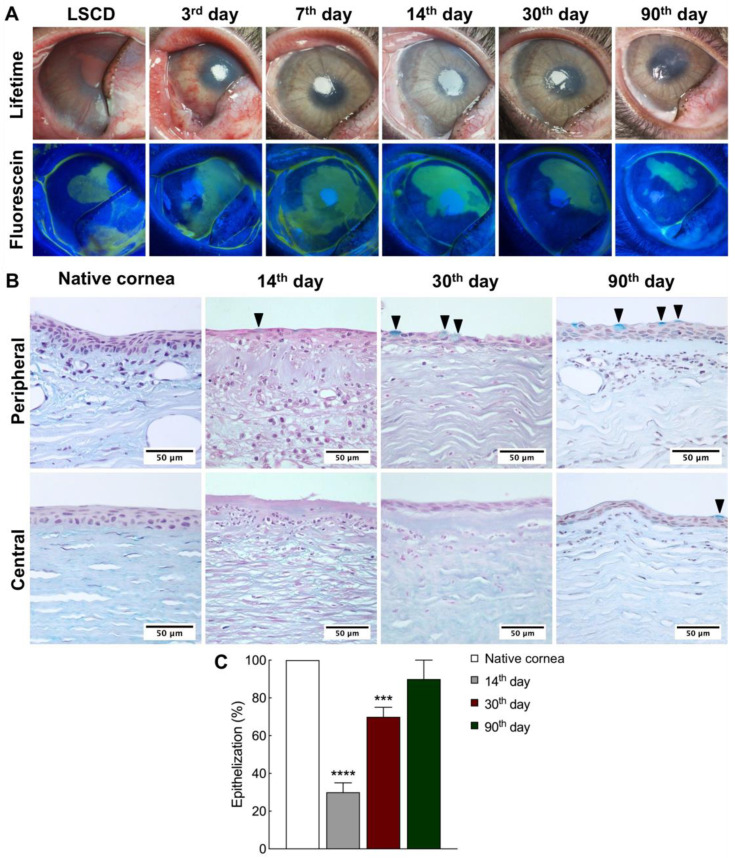
The rabbit cornea after transplantation of L-MSCs-EGFP onto the decellularized amniotic membrane and epithelialization dynamics at different time points. (**A**) Representative lifetime and sodium fluorescein staining images of rabbit corneas with limbal stem cell deficiency (LSCD) and on the 3rd, 7th, 14th, 30th, and 90th days after transplantation of the tissue-engineered graft. (**B**) Hematoxylin-eosin-alcian blue staining of central and peripheral corneal regions before debridement (native cornea) and after TEG transplantation. Black arrows—goblet cells. Scale bars: 200 μm. (**C**) The efficiency of corneal epithelialization at different time points after TEG transplantation (n = 3; *** *p* < 0.0002, **** *p* < 0.0001).

The transplantation of L-MSCs-EGFP seeded onto a decellularized human amniotic membrane (dHAM) was performed in the rabbit model of total LSCD. The recovery process was monitored for three months after transplantation (Figure 2A); for more photos, see Supplementary Figure S1. Neo-vascularization was detected in all experimental animals at all stages. Partial lysis of the graft was observed on the 7th day after transplantation. The dynamics of corneal re-epithelialization were analyzed with fluorescein staining. Partial corneal re-epithelialization was shown on the 3rd day in all animals. Transparency was restored and epithelialization was completed in some animals by day 90; in the other animals, the central cornea remained non-epithelialized. One-third of the animals had an intense inflammatory process during the experiment, transparency took longer to restore, and epithelialization was less effective, especially in the central part of the cornea.

### 2.2. Epithelialization Process during Corneal Reconstruction

A combination of alcian blue and hematoxylin and eosin staining was used to elucidate corneal structure after transplantation and reveal the presence of goblet cells specific to the conjunctival epithelium and absent in native corneal epithelium (Figure 2B).

On the 14th day, after removing the fibrovascular pannus and transplantation of the tissue-engineered graft (TEG), partial corneal re-epithelialization was observed. Conjunctival epithelium-containing goblet cells migrated over the corneal surface. The epithelialization rate on the 14th day after transplantation was around 30% of the total corneal area (Figure 2C). On the 30th day, the epithelialization reached 70%. On the 90th day, there was 90% epithelialization on average and was even complete in one rabbit. On the 30th and 90th days after transplantation, goblet cells were present mainly in the peripheral part of the epithelium. In the central cornea, the squamous epithelium was observed to contain 2–4 layers of cells.

### 2.3. Vascularization and Inflammation Processes during Corneal Reconstruction

**Figure 3:**
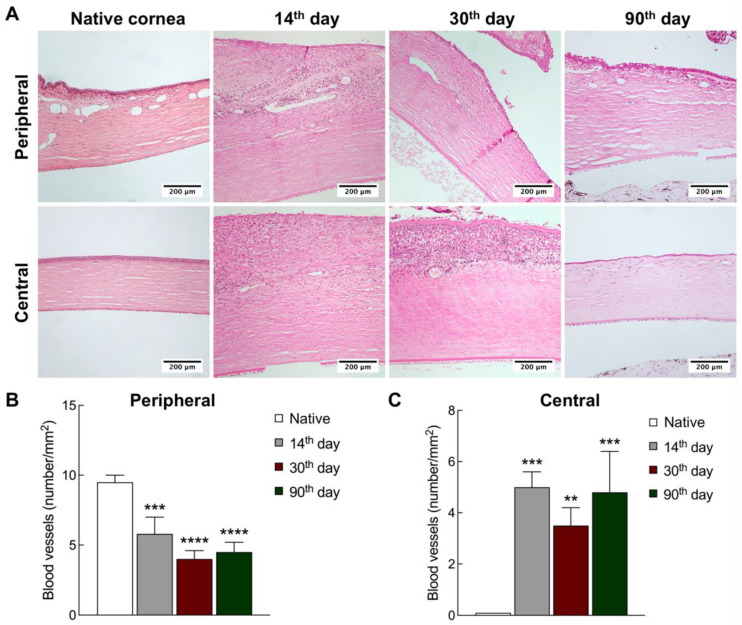
Corneal regeneration dynamics after transplantation of L-MSCs-EGFP onto the decellularized amniotic membrane. (**A**) Peripheral and central rabbit cornea at different time points after L-MSCs-EGFP transplantation. Hematoxylin-eosin staining. Scale bars: 200 μm. (**B**,**C**) The changes in the number of blood vessels in the peripheral and central stroma at different time points after transplantation (n = 3; ** *p* < 0.001, *** *p* < 0.0002, **** *p* < 0.0001).

The analysis of native corneas revealed only small venous vessels across the limbal region (Figure 3A), known to maintain the physiological functions of the residing LESCs [25]. After the TEG transplantation, venous vessels were replaced by capillaries in the limbal area, and capillary vessels formed in the corneal stroma. No inflammation was observed across native corneal tissues, while granulation tissue was formed beneath the TEG on the 14th day after transplantation. It should be noted that the presence of inflammatory cells (histiocytes, lymphocytes, and, to a lesser extent, eosinophils) was found across the corneal stroma at all time points. On the 90th day, mature connective tissue was formed under the epithelium throughout the cornea, and the inflammation significantly decreased.

A quantitative analysis of blood vessels in the limbal and corneal stroma was also performed at different time points after transplantation of the TEG (Figure 3B). The average amount of vessels in the limbal area of the native cornea was 9.5 ± 0.5 per 1 mm^2^, and there were no blood vessels in the corneal stroma. On the 14th day after the TEG transplantation, the number of vessels significantly decreased (5.8 ± 1.2 per 1 mm^2^) in the peripheral cornea and increased in the corneal stroma (5.0 ± 0.6 per 1 mm^2^) compared to the group of intact rabbits. On the 30th day, the number of vessels in the periphery of the cornea remained lower than in native tissue (4.0 ± 0.6 per 1 mm^2^) and preserved in the whole corneal stroma (3.5 ± 0.7 per 1 mm^2^). On the 90th day, the number of blood vessels within the peripheral area was also substantially decreased compared to the intact tissues (4.5 ± 0.7 per 1 mm^2^). In the corneal stroma, the vessels persisted, and their number was 4.8 ± 1.6 per 1 mm^2^.

### 2.4. L-MSCs-EGFP Localization in Corneal Tissue after Transplantation

The distribution and viability of green fluorescent protein-labeled cells in the corneal tissue were evaluated at different time points after the transplantation to rabbits.

**Figure 4:**
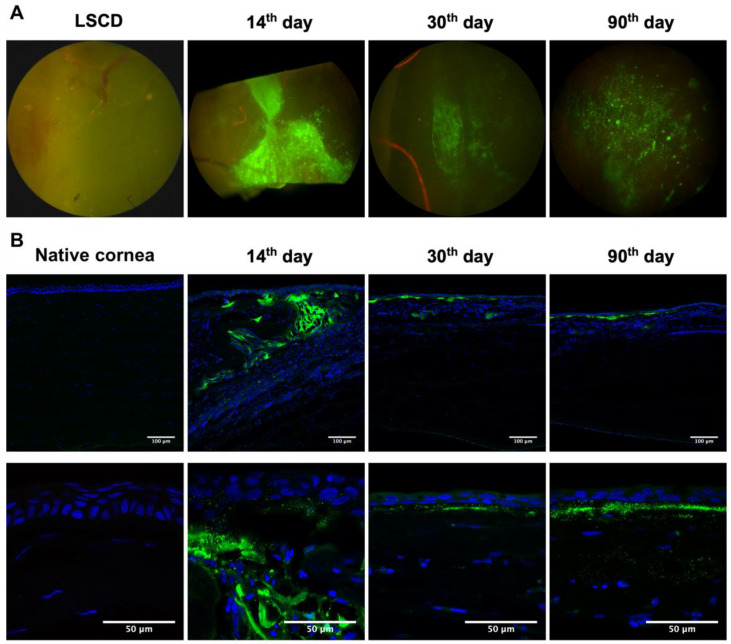
Localization of EGFP signal in rabbit cornea (peripheral area presented) at different times after transplantation of L-MSC-EGFP cells. (**A**) View from the top; corneal sectors with limbal stem cell deficiency (LSCD) and on the 14th, 30th, and 90th days after transplantation. Magnification ×50. (**B**) Localization of EGFP signal within rabbit corneal sections at different time points after L-MSCs-EGFP transplantation. Laser scanning confocal microscopy. Green—GFP channel; blue—DAPI. Scale bars: 100 μm and 50 μm.

The fluorescence of EGFP was detected at all time points after transplantation (Figure 4A). On the 14th day, the fluorescence was observed mainly in the peripheral corneal region. On the 30th and 90th days, weaker fluorescence was observed. The signal was present in both the peripheral and central corneal areas, though its localization varied in different animals.

Corneal sections were analyzed by laser scanning confocal microscopy on the 14th, 30th, and 90th days after the TEG transplantation (Figure 4B). On the 14th day, EGFP-labeled cells were presented in the corneal tissue and localized along the cornea’s periphery. L-MSCs-EGFP retained viability inside the folds of dHAM that were formed during its transplantation. The cornea was partially re-epithelialized by the conjunctival epithelium, and there were no labeled cells in the newly formed epithelium. Small clusters of L-MSCs-EGFP were detected in the central cornea. Fragments of destroyed cells or protein complexes were present in the stromal extracellular matrix. On the 30th and 90th days, particles with EGFP fluorescence were observed between the newly formed epithelium and stroma. No viable L-MSCs-EGFP were detected in the corneal tissue on these terms. On the 30th day, the squamous epithelium with 2–3 cell layers was formed over the particles containing EGFP. On day 90, the epithelium consisted of 3–4 cell layers.

**Figure 5:**
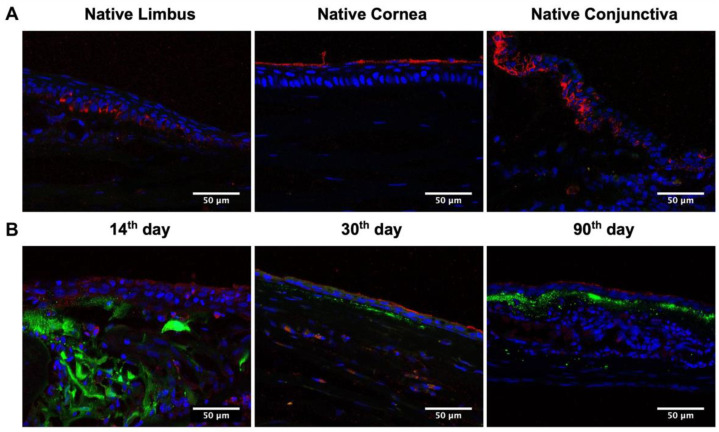
Immunohistochemical staining of rabbit corneas at different time points after transplantation of L-MSCs-EGFP. Red—staining with antibodies against cytokeratin 15; green—GFP channel; blue—DAPI. (**A**) Native tissue in the area of the limbus, cornea, and conjunctiva. (**B**) Corneal tissue on the 14th, 30th, and 90th days after transplantation of L-MSCs-EGFP. Laser scanning confocal microscopy. Scale bars: 50 μm.

Staining the native cornea with cytokeratin 15 (CK15) antibodies demonstrated the different patterns of CK15 in conjunctival, limbal, and corneal epithelia. CK15 was expressed in the basal and suprabasal layers of the conjunctival epithelium, the basal layer of limbal epithelium, and the superficial layer of the corneal epithelium (Figure 5). On the 14th day after transplantation of L-MSCs-EGFP, a weak CK15 signal was detected in all layers of the newly formed epithelium over the folds of dHAM containing the L-MSCs-EGFP. On the 30th and 90th days after the TEG transplantation, the CK15-positive cells were observed in the superficial layer of the squamous epithelium, like in the control sample.

## 3. Discussion

Recently, we demonstrated that rabbit L-MSCs had a high proliferative potential in vitro, expressed epithelial markers, and could differentiate into epithelial-like cells [22]. The ability of MSCs of various origins to differentiate in diverse directions has been widely discussed in the literature [14,17,18,26,27,28,29]. Several studies have shown the efficiency of MSCs for corneal epithelial repair in vivo [17,18,26,28,29] and during clinical trials [30]. The authors have considered the possibility of differentiation of MSCs into epithelial cells and the positive effect of paracrine factors produced by transplanted MSCs on corneal epithelial repair [29,30].

For reliable analysis of stem cell survival, growth, differentiation, and migration after transplantation, it is critical to track them in the host tissue [31]. Sánchez-Abarca et al. demonstrated that after subconjunctival transplantation of green fluorescent protein-labeled human MSCs to mice, the labeled cells were localized in the stroma and the corneal epithelium [28]. A study by Arnhold et al. demonstrated that green fluorescent protein-labeled MSCs could integrate into the retinal pigment epithelium and become hexagonal-shaped, which is typical for cells of this epithelium [32].

This study aimed to assess the fate of L-MSCs-EGFP [22] after transplantation onto a damaged cornea in the LSCD model in rabbits. Cells were transferred as part of a tissue-engineered graft based on dHAM. The amniotic membrane is the most widely used substrate for ocular surface reconstruction. It has highly biocompatible non-inflammatory qualities, may maintain corneal transparency, and promote cell adhesion, proliferation, and differentiation [33,34,35].

Herein, we demonstrated the following restoration processes in the corneal tissue after TEG transplantation: reduction of limbal vascularization, neoangiogenesis in the cornea, its epithelialization, and inflammatory infiltration. Similar results of corneal restoration using dHAM and stem cells have been previously reported [36]. We observed the migration of the conjunctival epithelium containing goblet cells over the corneal surface and assume its partial replacement by a corneal-like epithelium. By the 90th day, epithelialization of the rabbit cornea reached 90% on average, but no labeled cells were found in the newly formed epithelium.

Despite the attempt to create a physical barrier during the transplantation by forming dHAM folds, on the 14th day, cells that had migrated from the conjunctival epithelium were observed in the limbal area, and no labeled cells were found in the epithelium. A more effective physical barrier is needed to prevent re-epithelialization by the conjunctival epithelium. Nevertheless, the squamous epithelium with 2–4 cell layers and without labeled cells was observed on the 30th and 90th days after TEG transplantation. Even though CK15 is considered a limbal stem cell marker and is absent in differentiated human and mouse corneal cells [37,38], CK15 staining was observed in the superficial layer of newly formed squamous epithelium on the 30th and 90th days after TEG transplantation and showed a pattern similar to the control samples of native rabbit corneal epithelium. This means that the microenvironment of the corneal stroma may promote the differentiation of conjunctival cells and the formation of a squamous corneal-like epithelium [39]. Some biologically active factors and/or proteins secreted by transplanted cells could also have a positive therapeutic effect even after death.

On the 14th day, the labeled cells remained viable only at the periphery, where they were covered with dHAM folds and protected from mechanical damage. Insufficient mechanical protection and nutrient supply could cause the death of all other transplanted cells at early stages since the TEG was placed with the cells facing up. Zhurenkov K. and coauthors compared two approaches for the transplantation of TEG with labial mucosa stem cells seeded onto dHAM onto the rabbit cornea [36], with the cells facing up and down relative to the corneal stroma. The graft with stem cells-up was chosen according to the demonstrated efficacy of corneal restoration and epithelialization. In other studies, MSCs on scaffolds were transplanted onto the cornea with cells facing up and protected by tarsorrhaphy [40] or covered with a blank amniotic membrane [16]. Native amnion could provide mechanical protection and possibly secrete bioactive proteins that may promote more effective healing or stem cell differentiation [41]. Hence, such protection is essential, especially after the early stages of transplantation.

This study showed that no viable EGFP-labeled cells remained even in dHAM folds late after transplantation (on the 30th and 90th days). Therefore, other factors may lead to the death of transplanted cells. Although the cornea is generally considered an immunologically privileged organ because of its avascular structure, severe inflammation and neovascularization, specific to limbal stem cell deficiency, may result in an immune response to the transferred cells [42]. The authors also noted the low immunogenicity of MSCs of different origins, including L-MSCs [24]. Nevertheless, immunosuppressants are considered to preserve transplanted cells until complete regeneration and restoration of normal corneal barrier function [42].

Accompanying therapeutic agents, such as anesthetics, antibiotics, and anti-inflammatory drugs used during surgical manipulations and in the postoperative period, may also contribute negatively to the survivability of transplanted cells. The toxic effect of ophthalmic medications has been shown in vitro [43,44]. Assessing the toxic impact in vivo is still challenging. The selection of the optimal concentration and frequency of application for each therapeutic agent could positively influence the survival rate of transplanted cells and corneal epithelialization in general. In addition, an initial induction of epithelial differentiation in vitro also should increase cell survival and accelerate the epithelialization [40].

## 4. Materials and Methods

### 4.1. Cell Cultures

Rabbit L-MSCs with overexpression of green fluorescent protein (L-MSCs-EGFP) were derived, as previously described by Khorolskaya et al. [22]. Rabbit L-MSCs at 3rd passage were transduced with the lentivirus LV-CMV-EGFP Hygro (656-4) in Opti-MEM medium (Gibco, Waltham, MA, USA). During transduction, polybrene (Sigma-Aldrich, St. Louis, MO, USA) was used at a concentration of 8 μg/mL. Lentivirus LV-CMV-EGFP Hygro (656-4) was added at 5 MOI to rabbit L-MSCs and incubated for 20 h. Cells expressing EGFP were sorted by FACS using an S3e cell sorter (BioRad, Laboratories, Hercules, CA, USA) 72 h after transduction.

All cultures were maintained in DMEM/F12 medium (Gibco, Waltham, MA, USA) supplemented with 10% FBS (Gibco, Waltham, MA, USA), 1000 U/mL Penicillin/Streptomycin (BioloT, St. Petersburg, Russia) in 5% CO_2_ in a humidified incubator at 37 °C.

### 4.2. Animals

The work was performed on ten mature male Chinchilla rabbits (20 eyes were used in this study). All procedures were performed according to the rules of the treatment of laboratory animals confirmed by the certificate of OLAWNIH (Identification number F18-00380 of the Institute of Cytology of the Russian Academy of Sciences).

### 4.3. Corneal Graft Preparation

The human amniotic membrane (HAM) was used as a scaffold to make tissue-engineered grafts. HAMs were obtained during planned Caesarean deliveries from healthy patients (n = 5). All procedures were performed following informed consent of use for research purposes.

The L-MSCs-EGFP were seeded on the decellularized human amniotic membrane (dHAM) to make a TEG. For these purposes, HAMs were mechanically separated from the chorion, washed in Ringer’s solution containing ceftriaxone (10 mg/mL; Sintez, Moscow, Russia), fixed onto 3 cm Ø Petri dishes without bottoms with the epithelial side up under sterile conditions. Then, HAMs were cryopreserved at −80 °C in a mixture of DMEM/F12 medium (Gibco, Waltham, MA, USA) and dimethyl sulfoxide (1:1) (DMSO; BioloT, St. Petersburg, Russia). Before further use, dHAMs were thawed at 37 °C, washed 3 times with PBS, and decellularized at 37 °C for 45 min using 0.25% Trypsin-EDTA (BioloT, St. Petersburg, Russia).

Rabbit L-MSCs-EGFP were seeded onto the epithelial side of dHAM at a concentration of 1 × 10^4^ cells/cm^2^. TEGs were cultured for 3 days before transplantation to animals in DMEM/F12 medium (Gibco, Waltham, MA, USA) with 10% FBS (Gibco, Waltham, MA, USA), 1000 U/mL Penicillin/Streptomycin (BioloT, St. Petersburg, Russia), and 1% GlutaMAX (Gibco, Waltham, MA, USA) in a CO_2_ incubator under humidified conditions at 37 °C and 5% CO_2_.

### 4.4. Rabbit Limbal Stem Cell Deficiency Model and Graft Transplantation

All surgical procedures were performed under local anesthesia: local instillations of 0.5% Alcaine (Alcon, Geneva, Switzerland) followed by retrobulbar injection of 2% lidocaine solution (Renewal, Novosibirsk, Russia). Total LSCD was performed as previously reported [36] by removing limbal and adjacent corneal and conjunctival tissues (2 mm outside limbus, 4 mm in width, and 0.2 mm in depth). The remaining epithelium was scraped off the corneal surface. Moxiflox eye drops (moxifloxacin 5 mg/mL C.O. Rompharm Company S.R.L., Otopeni, Romania) and dexamethasone (1 mg/mL Belmedpreparaty, Minsk, Belarus) were applied after surgery and were then administered 6 times a day for 4 weeks after surgery.

The fibrovascular pannus developed on the rabbit eye 30 days after surgery and was separated and removed from the corneal stroma. The tissue-engineered graft (TEG) was placed on the prepared corneal surface. The TEG was sutured to the episclera with the cells facing up so that its folds formed a roller on the periphery of the cornea to perform a barrier function between the conjunctival epithelium and the corneal graft.

### 4.5. Cornea Regeneration Assessment/Graft Transplantation Assessment

The graft’s opacity, neovascularization, and epithelialization were assessed qualitatively using a surgical microscope (LOMO, Saint-Petersburg, Russia) on the 3rd, 7th, 14th, 30th, and 90th days after transplantation. To evaluate the dynamics of corneal epithelialization, rabbit eyes were stained with 1% fluorescein sodium solution (Novartis, Basel, Switzerland) and photographed under blue light.

### 4.6. Histological Analysis

The dynamics of corneal regeneration were assessed via histological evaluation of corneal epithelization, inflammation, and vascularization. The degree of inflammation was assessed qualitatively via the analysis of the presence of inflammatory cells in the corneal stroma. The degree of corneal epithelization was estimated by measuring epithelialized and non-epithelialized regions of the corneal sections using the ImageJ v.2.1 software. Corneal vascularization was estimated quantitatively via the analysis of the number of blood vessels per mm^2^ across the limbal region and corneal stroma.

Enucleated right rabbit eyes on the 14th, 30th, and 90th days after transplantation of TEGs were fixed in a 4% paraformaldehyde solution (PFA; Sigma-Aldrich, St. Louis, MO, USA) for 3 days. Afterward, the cornea and adjacent sclera were mechanically dissected, dehydrated, embedded in paraffin, and cut into 4 μm thick tissue sections. Tissue sections were stained with hematoxylin-eosin (Biovitrum, Saint-Petersburg, Russia) and alcian blue (Panreac, Barcelona, Spain). Sections were examined using Leica DMLS light-optical microscope (Leica Microsystems, Wetzlar, Germany).

### 4.7. Analysis of Localization of EGFP-Labeled Cells within Rabbit Cornea

The viability and distribution of EGFP-labeled cells in the corneal tissue were evaluated on enucleated left rabbit eyes on the 14th, 30th, and 90th days after transplantation of TEGs. Eyes were fixed in 1% PFA for 3 days. The cornea and adjacent sclera were separated from the rest of the eye tissues. The surface was analyzed by fluorescence microscopy in the GFP channel using a Leica DM6000B fluorescent microscope (Leica Microsystems, Wetzlar, Germany). Then, the corneas were incubated in 20% sucrose solution with 1% PFA for at least 24 h. After that, the samples were embedded in Tissue-Tek O.C.T. Compound (Sacra Finetek, Torrance, CA, USA) and frozen for 2 h at −23 °C. Cornea cryosections that were 12 µm thick were obtained using a Leica CM-3050S cryostat (Leica Biosystems, Deer Park, IL, USA). Cell nuclei were stained with DAPI (1 µg/mL; Thermo Fisher Scientific, Waltham, MA, USA). Visualization was performed using single scans made using an OLYMPUS FV3000 confocal microscope (Olympus, Center Valley, PA, USA). Autofluorescence was filtered out during image acquisition and normalized according to the level of autofluorescence on the slides with native cornea. ImageJ (v.2.1 software) was used to process and analyze the obtained images.

### 4.8. Immunohistochemistry

The corneal cryosections were permeabilized with 0.1% Triton X-100 (Sigma Aldrich, St. Louis, MO, USA) for 15 min and blocked in PBS supplemented with 10% FBS (Gibco, Waltham, MA, USA) and 1% bovine serum albumin (BSA; Thermo Scientific, Waltham, MA, USA) at 37 °C for 1 h. Hybridization with primary antibodies against Cytokeratin 15 diluted to 1:500 (MA5-15567; Thermo Fisher Scientific, Waltham, MA, USA) was performed overnight at +4 °C. After several washes, the Anti-Mouse IgG H&L antibodies conjugated with goat anti-mouse Alexa Fluor 555 diluted to 1:500 (ab150114; Abcam, Cambridge, UK) were added for 60 min at room temperature. The nuclei were counterstained with DAPI (1 µg/mL; Thermo Fisher Scientific, Waltham, MA, USA). Sections were observed using single scans made using an OLYMPUS FV3000 confocal microscope (Olympus, Center Valley, PA, USA). ImageJ (v.2.1 software) was used to process and analyze the obtained images.

### 4.9. Statistical Analysis

All statistical calculations and graph plotting were performed using Prism 9.0 (GraphPad Software, San Diego, CA, USA). In all experiments, at least three independent measurements were performed. Error bars represent the mean’s standard deviation (S.D.), analyzed a priori for homogeneity of variance. Differences between groups were determined using a one-way analysis of variance (ANOVA) followed by Dunnett’s multiple comparison post hoc test. Significance between groups was established for *p* < 0.001, *p* < 0.0002, and *p* < 0.0001 with a 95% confidence interval.

## 5. Conclusions

Labeled stem cells are a valuable tool to evaluate their role in restoring host tissue and clarifying the underlying mechanisms of the impact of transplanted stem cells on regeneration. They allow us to evaluate the migration and survival rates of the transferred cells and therefore are necessary for selecting optimal conditions for stem cell transplantation.

This study demonstrated the low survivability of L-MSCs-EGFP transplanted as a part of TEGs. Presumably, under the given transplantation conditions, without additional mechanical protection, in acute inflammation and under the influence of adjuvant therapeutic agents (local anesthetics, antibiotics, and anti-inflammatory agents), the transplanted L-MSCs-EGFP insufficiently survived to restore the damaged limbal niche. However, the fragments of destroyed cells or protein complexes containing EGFP were preserved in the corneal tissue even on the 90th day. It is possible that paracrine secretion of transplanted cells may have a positive therapeutic effect, but this assumption still needs to be clarified. The selection of optimal conditions and therapeutic medications, including immunosuppressive drugs, is one of the aspects we need to improve for further studies of the regenerative potential of L-MSCs-EGFP or any other stem cell types.

This study provides a better understanding of the processes occurring during the transplantation of TEGs onto the corneal surface. It paves the way for further analyses of their participation in corneal regeneration.

## Data Availability

Not applicable.

## Supplementary Materials

The supporting information can be downloaded at: https://www.mdpi.com/article/10.3390/ijms24065431/s1.
