# Supplementary material for: Corneal Reconstruction with EGFP-Labelled Limbal Mesenchymal Stem Cells in a Rabbit Model of Limbal Stem Cell Deficiency
Source: Int J Mol Sci. 2023 Mar 12;24(6):5431. doi: 10.3390/ijms24065431 (PMC10051408; doi:10.3390/ijms24065431)
Supplement: Supplementary file 1 [file ijms-24-05431-s001.zip › ijms-2230478-supplementary.pdf]

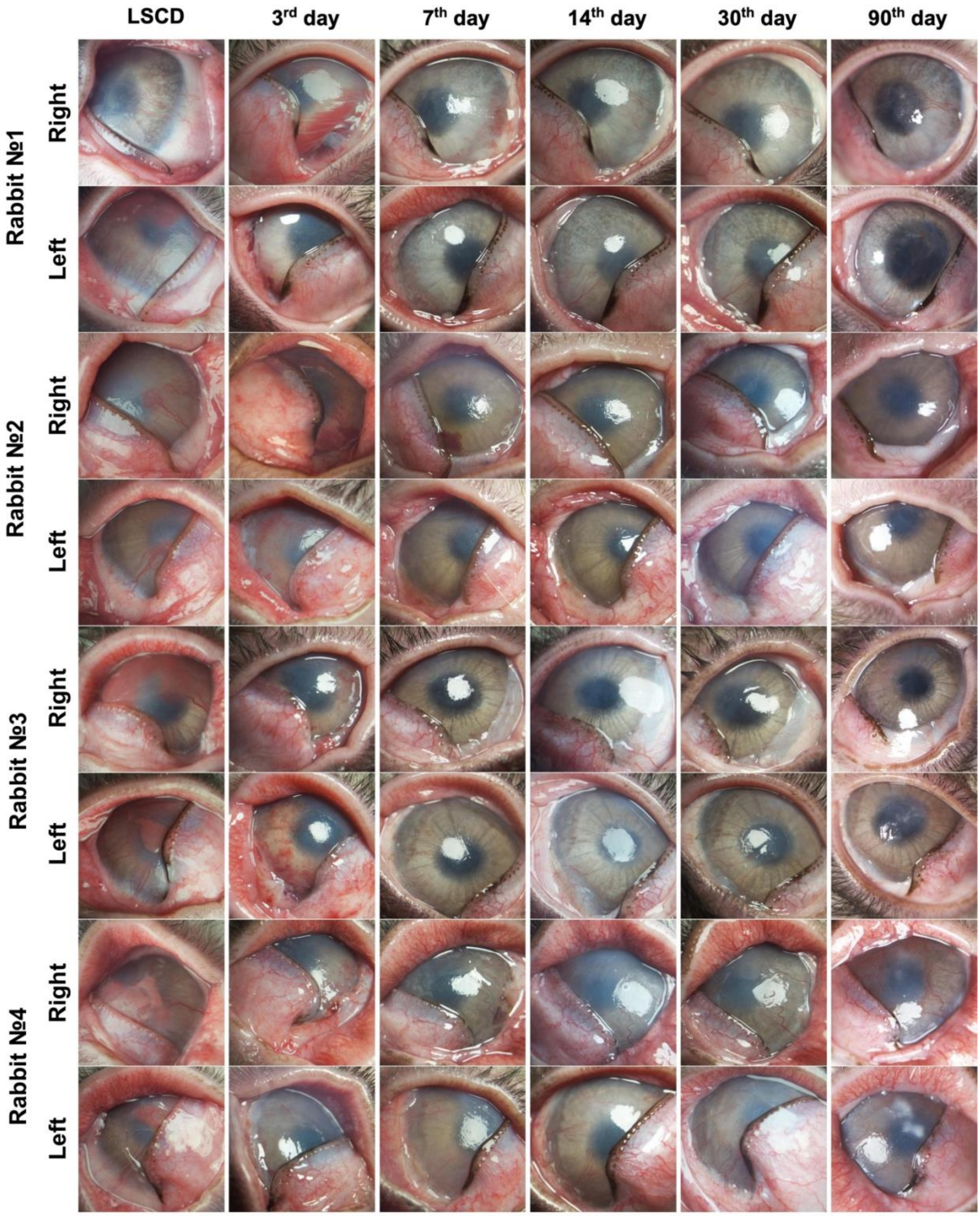

Figure S1. The rabbit cornea after transplantation of L-MSCs-EGFP onto the decellularized amniotic membrane at different time points.
